# Pre- and Post-Migration Influences on Weight Management Behaviours before and during Pregnancy: Perceptions of African Migrant Women in England

**DOI:** 10.3390/nu13051667

**Published:** 2021-05-14

**Authors:** Lem Ngongalah, Judith Rankin, Nicola Heslehurst, Tim Rapley

**Affiliations:** 1 Population Health Sciences Institute, Newcastle University, Newcastle upon Tyne NE2 4AX, UK; judith.rankin@newcastle.ac.uk (J.R.); nicola.heslehurst@newcastle.ac.uk (N.H.); 2 Department of Social Work, Education and Community Wellbeing, Northumbria University, Newcastle upon Tyne NE7 7XA, UK; tim.rapley@northumbria.ac.uk

**Keywords:** diet, physical activity, weight management, pregnancy, African migrant

## Abstract

The prevalence of overweight/obesity is high among Black women in England, who also face high risks of pregnancy and childbirth complications. This study explored African migrant women’s perceptions of pre- and post-migration influences on their weight-related behaviours and weight management support during pregnancy. Interviews were conducted with women of child-bearing age from Ghana, Nigeria, and Cameroon (*n* = 23). Data were analysed using thematic analysis. Four themes were identified: changing dietary behaviours after migration, changing physical activity (PA) behaviours after migration, increased discourse on obesity, and weight management advice and support received. Navigating a new food environment, interactions with other populations in England, and the need to socialise influenced changes in dietary behaviours. Participants considered that living in England ‘makes you lazy’ due to its obesogenic environment, while increased discourses on obesity heightened weight awareness. Women struggled to relate to dietary advice from midwives but found PA advice useful. Relatives provided valuable support but could influence unhealthy weight-related practices. There is a need for interventions addressing gaps in weight management support for these women, especially considering their migrant backgrounds and multicultural identities. Further research is needed to understand their unique challenges, and collaborations with relatives could inform the development of effective weight management interventions.

## 1. Introduction

There is a higher prevalence of overweight and obesity among Black women in England compared to women from other ethnic groups [1]. Black women are also more likely to die from complications during pregnancy and childbirth [2]. Overweight and obesity during reproductive age predispose women to adverse perinatal outcomes including stillbirths, caesarean delivery, perinatal mortality, gestational diabetes, and pre- and post-term births [3,4,5,6,7,8]. Ethnic disparities in these outcomes are evident in England, with Black women bearing greater risks [1,2,9].

Behavioural factors such as diet and physical activity (PA) are amongst the wider determinants of weight status in women of child-bearing age [10,11]. UK guidelines recommend that women need to be supported before conception and throughout pregnancy to have and maintain healthy dietary and PA behaviours, in order to improve their pregnancy outcomes and optimise the health of their children [12]. There are various guidelines and resources available to support the dietary and PA behaviours of pregnant women and women of child-bearing age in England, such as healthy eating guidelines provided by the NHS [13], weight management advice provided by midwives or health visitors [12], PA infographics for pregnant women and women of child-bearing age [14], and other weight management programmes such as aquanatal classes, park walks, and cooking programmes [15]. While several studies have explored women’s experiences and perceptions of weight management support in England [16,17,18,19], little is known about the experiences of women who have migrated to England from Africa (henceforth referred to as African migrant women). In addition, dietary guidance in England is usually centred around traditional British foods and cooking/eating patterns, which may differ from those of African migrant women.

Dietary and PA behaviours can be influenced by individual ideologies of appropriate behaviours, and they can also be reinforced by social, cultural, and environmental factors [20]. The experience of transitioning from one country to another exposes migrant women to different cultures and environments, and this process plays a role in determining their weight perceptions and related behaviours [21,22]. Migrant women’s behaviours can also be shaped by their relationships with their societies both before and after migration [23,24,25]. Pregnancy can also be particularly challenging for migrant women, not only due to the associated physiological changes, but also as a consequence of their changed environments, identities, and social networks [26]. These changes may influence their weight-related behaviours and needs in pregnancy.

We previously published a systematic review on the dietary and PA behaviours of African migrant women living in high-income countries (HICs), which showed the adoption of bicultural dietary patterns after migration, nutrient inadequacies, and reduced PA levels [27]. The present study aimed to explore the lived experiences of African migrant women in England, to gain an in-depth understanding of the pre- and post-migration influences on their weight-related behaviours, including an exploration of their perceptions on the support they received to manage their weight during pregnancy.

## 2. Methods

### 2.1. Study Design and Context

This was a qualitative study which took place in England. Participants were African migrant women who had migrated from three countries in sub-Saharan Africa—Cameroon, Ghana, and Nigeria. Nigeria and Ghana were selected because these are amongst the largest African migrant populations in England [28], while Cameroon was selected due to the principal investigator’s links and access to this community of migrants in England.

### 2.2. Participant Recruitment

Eligible participants were women aged 18–45 years, who had lived in England for at least 6 months, and were either currently pregnant or had given birth within the last 3 years. Participant selection involved a combination of purposive, convenience, and snowball sampling methods. Women were purposively targeted through community groups or organisations serving African populations in England, such as churches, nongovernmental organisations, and support groups. A flyer containing information about the study and researcher contact details was shared with these organisations, and interested participants then made contact. Participants were also targeted through social media; the study details and contact information were circulated through Facebook, WhatsApp, and Twitter, to reach African groups online. Women were also recruited using convenience sampling through personal networks and snowball sampling through referrals.

### 2.3. Data Collection

Data for this study were collected through semi-structured interviews, conducted by L.N., either face-to-face or over the telephone. In-person interviews took place in locations where participants deemed convenient, such as at their homes or in quiet public spaces. Participants provided written consent before each interview, while those who were interviewed over the telephone provided consent by email and verbally. A flexible topic guide was used during interviews (Appendix A). The topic guide consisted of six main questions, with follow-up points listed below each question to address key issues. Interview questions were open-ended, to encourage discussion. The topic guide was piloted before data collection and refined throughout the course of the interviews, to further explore emerging themes, as data analysis commenced during data collection.

Interviews were carried out from November 2018 to May 2019. These were recorded using an encrypted digital recorder, and the data were stored on a secure network at Newcastle University. All data storage arrangements were made in line with the Newcastle University Information Security Guidelines [29] and the General Data Protection Regulation [30].

### 2.4. Data Analysis

All interviews were transcribed and anonymised. Participants were assigned an identification number (e.g., P1) to preserve anonymity. Data were analysed using thematic analysis, following the guidelines provided by Braun and Clarke [31]. This process involved reading the transcripts repeatedly to become familiar with the data, assigning initial codes to emergent ideas, and finally grouping similar codes into themes. Data coding was supported through using NVivo-12 [32]. The various stages of the thematic analysis process were repeated until no new codes or themes emerged from the data. Resulting themes and subthemes are presented in Section 3. Participant identification numbers are provided for longer person-specific quotes, while commonly held views or terms used by many women are not tied to any identification numbers. Pauses are indicated using an ellipsis (…) and quotation marks are used for specific terms used by participants. Additional information provided for clarification and context is shown in square brackets. Illustrative quotes are provided.

A conceptual framework was developed from the study results using the socioecological model of health [33], to show the interactive influences of pre- and post-migration factors on women’s weight-related behaviours. This model considers the complex interplay of individual, relationship, community, and societal factors on health outcomes and behaviours. It recognises that multiple levels of influence exist, and also that these levels are interactive.

## 3. Results

Twenty-three women participated in this study (Table 1). Participants were aged 23 to 41 and had lived in England for an average of 6.8 years. Most of the women were either married or cohabiting (*n* = 19), had two or more children (*n* = 15), had studied to university level or higher (*n* = 16), and were employed (*n* = 17). Five women were pregnant at the time of interview, three of them being pregnant for their first time. Nine women had given birth in the last year, five had children aged 1–2 years, and four women had children between 2 and 3 years of age.

### 3.1. Summary of Themes

Four overarching themes resulted from this study: (a) changing dietary behaviours after migration, (b) changing PA behaviours after migration, (c) increased discourses on body weight and obesity, and (d) weight management advice and support received.

- Changing dietary behaviours after migration

Three subthemes emerged from the data on dietary changes, which were (i) navigating a new food environment, (ii) the need to socialise, and (iii) changing attitudes towards food labels and taboos.

(i) Navigating a new food environment

Participants viewed unhealthy dietary behaviours such as ‘eating junk food’ as dominant causes of overweight and obesity. However, despite holding these views on health risks, they described how they frequently consumed ‘junk foods’, especially in their early months/years of arriving in England. The term ‘excitement’ was used commonly when describing their eating behaviours during this period—excitement of having migrated to a new society where there is an abundance of food items that were either expensive or not commonly eaten in their countries of origin. Examples included chicken which is ‘more expensive than fish and beef back home’, burgers which are ‘not very common’, and pizza which is ‘quite expensive’.

The women gave various reasons for visiting fast-food shops frequently. For example, they ‘enjoyed’ the taste of chicken and chips, mainly because it was prepared differently from what they were accustomed to back home (e.g., adding batter). They also reported being attracted by the smell from fast food shops when walking by, which they found to be ‘very tempting’. One participant reported that this was ‘because I was yet to figure out where to buy raw food that I can cook’ (P9), while another related this to the ‘bad UK weather’ (P8) and close proximity of fast food shops to her house:

I came here around November and the weather was horrible. Horrible! You know when you just came from that hot, nice weather back home… it’s like now you live in a, in a freezer. For me to go out and go to the market or to go and buy food, it was difficult. Walk like 10 or 15 min in that cold? I couldn’t. There was one ummm… one chicken and chips shop just opposite my house, many of them even, but one that I liked a lot. So, it was easy for me to just jump in there, get my food, and jump back out (P8).

This quote shows an example of the obesogenic nature of the English environment. Other women shared similar stories relating to their places of work, universities, or colleges. The close proximity of fast-food outlets to women’s environments contributed to an increase in fast-food consumption among some of the women interviewed. Over time, however, participants consumed fast food less frequently because they were ‘getting tired of it’ (P4), ‘missed the taste of African food’ (P13), or ‘started realising that fast food is expensive’ (P1). Some participants felt that they had suddenly gained a lot of weight, which to them was an indication that ‘too much junk food is not good for you’ (P7). This led the women to start thinking about where to buy food, what kinds of food to buy, and where they could find African food shops.

Two factors that influenced participants’ dietary behaviours were where they lived and who they lived with. One participant shared her experience of living in a household where traditional African dietary preferences were preserved:

I came as a student and I was living with my brother. His wife was always cooking African food so for me… I didn’t really feel like I miss anything. Even though, the African food here does not really taste the same as back home because, you know, there are some… ummm, some spices that you’ll never find here (P13).

In this example, the participant mostly consumed African food, albeit lacking a sense of authenticity due the absence of some ingredients. In contrast, another participant shared how she ‘had to’ adopt a Western dietary pattern due to the dietary preferences of the relatives with whom she lived:

All these lasagne, jacket potato, baked beans… even the way they made pasta was different. They tried to make me like them, but those things are not for me. But I can’t really say anything because… well, I’m staying in someone’s house and they’re kind to take care of me. Until I can work, start working, and move to my own house before I could, I can really decide what I like to eat (P9).

Her example demonstrates her relative lack of autonomy to make decisions about her food consumption, as she had little choice but to follow an acculturated dietary pattern. Thus, the decision to maintain or change dietary behaviours was sometimes influenced by factors beyond personal choice. Other participants described having ‘switched’ to British or ‘Western’ dietary behaviours due to various factors, including who they socialised with (e.g., having mostly White friends), living in predominantly White cities where there were ‘very few or no African food shops’ or markets, deciding to ‘try new things’, or ‘because I’ve been eating African food my whole life so I wanted a change’ (P19). Meanwhile, living in cities like London and Birmingham where ‘there are many African shops’ was an enabler for preserving the African dietary pattern, although African food in England was usually very expensive, ‘sometimes even more than ten times the cost back home’ (P14). Participants also felt that African food bought from England did not always taste the same as the food they knew back home. As such, to enable them to preserve their traditional food patterns, the women reported having to ‘pay for African food to be sent from back home’ (P2) or ‘carry bags of food from home and bring here whenever you travel’ (P17).

(ii) The need to socialise

Eating African food was seen as a way of maintaining cultural identity, and many women stated that they followed a ‘strictly traditional African’ dietary pattern. A common phrase used when talking about preserving their ethnic food culture was that ‘it’s important to always remember your roots’. Expanding on why this cultural food identity was so important to them, women talked about the nostalgic remembrance of back home when eating African food, which also invoked happy memories of being with family and loved ones:

When you eat African food… especially if you’re eating with other people, you remember how you used to eat back home as family, and sometimes it reminds you of some nice stories, you know… sometimes I like to call my mom when I’m eating, and she’ll be happy that at least I can still eat the food I like even here (P11).

Preserving African dietary patterns is shown here as a way of connecting with family and recollecting fond memories of back home. Some women felt that these memories helped to distract them temporarily from the ‘harsh’ realities or stress they were experiencing as migrants in their new living environments. On the other hand, participants also felt a need to feel some form of identification with other groups of people in their new society, in order to feel like they fit in or belonged in their social circles:

As a Nigerian, you want to stick to your Nigerian food and eat the things you’ve been taught to eat from childhood… but then you think again, you think of yourself being a foreigner staying in England… you want to also, you want to adapt to the society you’re in, because I mean you’re probably working or schooling or making friends with people who are not African. How do you socialise with them if you cannot eat together? (P14)

The ability to eat other kinds of food is shown here as enabling for social life. Hence, the women’s dietary behaviours evolved as they interacted with others in England. Thus, irrespective of personal will to preserve ethnic food practices, there was always some level of in betweenness as these migrant women integrated into their new societies. In another response, a participant described making efforts to identify with other people in her social circle:

You can’t really bring them to an African restaurant, you’re not sure if they’ll even know, talk less of liking anything there. Even if you succeed to convince one or two people to… to try out an African dish, it’s only fair that you be open to eat their own food as well. So, whether you like it or not, sometimes you have to go with them. You go to an English restaurant or Italian, or fast food… it’s part of socialising. You can’t always be the one to choose where to eat, and you can’t be picky every time or turn down invites to hang out… that will not look nice of you (P12).

She demonstrates, here, the importance of reciprocity in establishing social relationships, and how there has to be a mutual exchange of actions between people. In this sense, the ability to socialise with other people sometimes required self-sacrifice, in that the women had to learn to eat what others eat. This shows that the women’s dietary practices became embedded (to varying extents) in the host country culture over time.

(iii) Changing attitudes towards food practices and traditions

Participants reported increased intake of fruits, vegetables, and water after migrating to England, which they consumed even more during pregnancy. The women described how it’s very common to find women in England trying to ‘watch what they eat’, which contrasts behaviours of African women whose food preferences are usually driven by taste. However, despite initial surprise, some participants eventually developed the habit of reading food labels, as well as calculating the caloric content of food before buying:

They read labels on food, they calculate calories… we don’t usually have time for that! We just buy and go. If it tastes good, then it’s good. It used to surprise me how people take time to do all those things… but after some time, I, I saw myself doing it. Now I check everything and I’m very careful with what I eat (P2).

Several women described similar experiences, for example, adopting the habit of checking whether food was organic or not, checking the percentage of fat or sugar, and making decisions about different kinds of milk. These behaviours, they noted, were usually influenced by interactions with White people or with Black people who were born in England. The women also reported having learnt to pay more attention to what they ate during pregnancy while being in England, as a result of being more exposed to health messages about nutrition in pregnancy. Examples of dietary changes made during pregnancy included reducing intake of ‘junk food’, drinking ‘a lot of water,’ avoiding food that is ‘too hot or too cold’, and substituting unhealthy cravings or snacks with fruits. Meanwhile, ‘in Africa, when you’re pregnant, there’s nothing like regulating what you eat’ (P9), because ‘that’s when you’re allowed to eat whatever you want’ (P13). The only exception to this notion of pregnant women eating anything they wanted was that some cultural food traditions prohibited pregnant women from consuming certain types of food:

They say if you eat eggs maybe you cannot have children again in the future. Then snails… because the child can be lazy (P3).

Other women described similar cultural taboos relating to food items such as beans, milk, honey, and ‘certain types of meat’. While living in England, participants started identifying some of these cultural food taboos as potentially harmful, especially because ‘sometimes it involves things are actually good for pregnant women’ (P10). In some cultures back home, pregnant women were also encouraged by relatives to take herbal medicines or drink traditional concoctions for various reasons, e.g., ‘to keep her and the baby healthy’ (P11) or ‘to prevent pregnancy sickness’ (P4). While these traditions were usually followed strictly back home, the women started questioning the content of these traditional medicines and concoctions after migrating to England and wondered if they were safe during pregnancy:

Me I don’t follow all those things. If you have headache or nausea, vomiting, anything, they’ll give you herbal drink and all sorts. We don’t even know what they mix inside… or whether it’s really safe or not. But since that I’m here, I think I’ve learnt more about what… to be careful with what I’m eating or drinking when I’m pregnant’ (P17).

These quotes show that women’s cultural beliefs were challenged after migrating to England, which could then contribute to changes in their weight-related behaviours. Living in England is also identified as a facilitator for healthy dietary behaviours, through increased exposure to healthy diet messages, which could also contribute to an increased personal awareness of healthy food practices both before and during pregnancy.

b. Changing PA behaviours after migration

Findings influencing women’s PA behaviours after migration were clustered into two subthemes: (i) living in England ‘makes you lazy’: the influence of the obesogenic environment, and (ii) changing weight stereotypes.

(i) Living in England ‘makes you lazy’: the influence of the obesogenic environment

In discussing factors associated with changes in their PA behaviours after migration, women frequently made statements such as ‘living here makes you lazy’ and ‘it’s very easy to get fat here’. Participants referred to the environment in England as obesogenic, noting that ‘here you live the easy life’, whereas the lifestyle back home is ‘more active’. Various examples were cited to illustrate this obesogenic environment, such as overreliance on mechanised transport, less outdoor activity due to bad weather, and less household work (both in amount and intensity) due to access to household appliances:

Here, we have hoovers, mops, washing machines, and all that to facilitate your work, so it doesn’t take long to clean. But, in Africa, first you have to bend down to sweep with the local broom, bend down to put the rag in water, squeeze and mop, and sometimes you even have to go on your knees to scrub the floor (P5).

This participant describes women being ‘more active’ in Africa because tasks like house chores are usually done manually, often on a daily basis. Women also compared the types of work-related activities that women do in Africa, which they described as ‘more energy-demanding’ than in England. For example, farming is commonly practised by many women in Africa, and these farms are sometimes very far from women’s homes, requiring that they walk long distances. They contrasted this with the lack of farming in England, where ‘you can only go as far as doing a bit of gardening near your house if you want to grow a few crops’ (P4).

However, the image of people back home being ‘very active’ was not consistent, as women also noted that ‘things are changing’ and that many parts of Africa—especially urban areas—are starting to be ‘a lot like some developed countries’. For example, walking long distances (e.g., to the market or farm) which used to be practiced frequently is now almost non-existent in some African communities, due to the increased use of cars and the availability of motorcycle taxis which are ‘cheap’ and ‘always available’:

You find them [motorcycles taxis] everywhere, and the cost to the market and back is not even up to 50 p. They’re also very helpful because they can reach places that cars cannot reach, like inside the quarter, so you don’t have to walk (P13).

As she described, these motorcycles had the added advantage of being able to travel roads without tracks which cars could use. This quote contrasts the finding that women were ‘very active’ back home. In discussing reasons associated with the changes in PA patterns back home, some women identified having lived in a developed country as having an influence on their commuting preferences when they returned to their home countries:

When I came to England, I became used to driving everywhere, you know, or riding the bus or the train… so, now when I go home, you will rarely see me walking. If I need to go even to the corner shop, I’ll just get in my car and drive. I always go to the market with either my mom or my sisters—my siblings, so now they also do same: drive or call a bike (P14).

Having lived in England in this example influenced both her commuting behaviours and those of her relatives. Other women cited similar examples of how they influenced the behaviours of their relatives back home, e.g., by buying and shipping hoovers and washing machines back home ‘to ease their work’, or by introducing their relatives to preparing and eating convenience foods ‘to save time’. Hence, having lived in a developed country is shown here as playing a negative role on the PA levels of participants and their relatives back home.

While noting the changes in PA as reported above, several participants interpreted PA as referring solely to sport or exercise, which, in their societies back home, were mainly regarded as activities for males or athletes. Women were also positioned as rarely engaged in such activities because they were usually busy with their multiple daily tasks and ‘because we always have other important things to do’ (P3). One participant noted that ‘even those who do sports do it because they want to, and ‘not because anybody is encouraging them to do it’ (P11). Hence, PA (or sport to some women) was not considered an important determinant of weight status for women of child-bearing age, since it was not an inherent part of their culture.

(ii) Changing weight stereotypes

Participants described how they felt it was very common to find women talking about ‘watching their weight’ in England, which contrasts the situation back home where it is believed that an African woman should be ‘thick and curvy’. As such, deliberate acts of weight loss that aim for a small body size were seen as relatively unthinkable:

Maybe they haven’t seen you for some time, then they see you and you look a lot smaller than the way you were, sometimes they’ll just believe you have HIV and you’re dying. They don’t think that someone can just want to lose weight like that (P1).

The women also identified that what they formerly considered ‘too small’ or ‘skinny’ back home was instead seen as ‘fit’ or ‘nice’ by people in England, which influenced them to desire smaller body sizes and engage in activities aimed at losing weight. Associating with White people or with Black people who were born in England was a key influence on these changes in weight perceptions: ‘When you’re always around… especially White people, you start to be more careful about gaining too much weight (P10). On the other hand, being routinely around other Africans was cited as a factor that ‘can easily influence a woman to think that she needs to add weight’ (P8):

When you’re always around Black people, it’s very easy for you to keep thinking that a woman should be… big, you know, bulky… and you can easily get fat and be overweight (P10).

While acknowledging the positive influence of living in a society where women were less stigmatised for being small in size, participants also reported feeling ‘uncomfortable’ with the ‘general notion most people have in England, that being fat means you’re unhealthy’ (P10). This shows that these women had experienced different forms of weight stigma, due to the different weight-related views in the two societies.

c. Increased discourses on body weight and obesity

Migrating to England was shown to have a positive influence on the women’s weight-related knowledge in pregnancy. Participants described how they felt that African women are usually ‘not bothered’ or less concerned about their weights, ‘except when they are pregnant’ (P8) or when their body sizes do not conform with societal preferences (e.g., when they believe they are ‘too fat’). Most women interviewed either believed that a woman’s weight status before pregnancy has ‘no effect’ on her health or were unsure about any health implications:

I don’t think being overweight or obese before you’re pregnant can affect in anyway… I’m not sure. I’ve not heard anything like that (P1).

Similar responses were shared by other participants, with a few women mentioning having heard ‘something like that’ in England, regarding the negative health consequences of preconception overweight or obesity. Meanwhile, some women questioned this information, believing that ‘it has not been proven’ (P5). As such, having an overweight or obese BMI before pregnancy was not considered as important a pregnancy risk as other factors like smoking and alcohol consumption, which participants had heard ‘so much’ about:

We hear a lot about um… smoking, and ummm alcohol, and… also drugs, taking drugs… because they’re very bad for you—for the mother and also the child. I think they’re the most important things that pregnant women should ummm, should be careful about (P2).

Like this participant, most women mentioned having heard ‘so much’ about the impacts of having a poor diet (e.g., low intake of fruits and vegetables, high intake of sugar, salt, and processed food), as well as smoking, consuming alcohol, and taking drugs during pregnancy, and that these could ‘harm the baby’, ‘can cause you to miscarry’, or ‘make you have high blood pressure’. Hence, these were believed to be priority risks for women. While they considered weight status to be important, it was not seen as a priority.

Having a greater exposure to health messages about weight status and the risks of excess weight gain while living in England contributed towards the women becoming ‘more conscious’ about their weights. For example, one participant recounted her experience of getting to know her BMI for the first time:

When you go to see the midwife, first thing is to take your weight. Yeah… it was, for me it was my first time to know about my BMI and I was surprised she said I’m a bit, a bit overweight (P2).

That she described being surprised to hear that she was overweight implies that she had a different perception of her weight status prior to this interaction. In another example, a participant described how she used to think of herself as being ‘skinny’ while living in her country of origin, and that this perception would have influenced her to make efforts to gain weight:

If I was back home now, like, I will be trying to gain weight [laughs], but here now I know that I’m not skinny like I used to think… so I don’t need to eat more, or try to… um… put on more weight (P9).

These two examples show how women’s perceptions of their own weights were different in the two societies where they have lived. The second quote also shows how the participant’s new weight perception eliminated the perceived need to gain weight. Thus, migrating to England influenced a change in her weight perception, which in turn had a positive influence on her weight management efforts.

While most participants in this study were familiar with the term BMI and acknowledged having talked about it with their health providers, they had different interpretations of its meaning. For example, BMI was thought to be ‘the difference between your weight before and after pregnancy’ (P8), ‘a given weight that should be attained at every age’ (P2), or ‘an average weight that should not be exceeded’ (P1). These findings highlight a knowledge gap for some of these women, which could potentially be an important step towards raising obesity awareness and weight management support for women of child-bearing age.

d. Weight management advice and support received

Three themes resulted from findings on weight management advice and support in pregnancy, which were (i) unrelatable advice from midwives, (ii) pregnancy pampering, and (iii) conflicting information on PA.

(i) Unrelatable advice from midwives

Participants noted a difference in the amount of diet-related advice provided to pregnant women in England compared to back home, highlighting that there is ‘a lot more information in England’. However, while acknowledging midwives as being relevant sources of diet-related advice in pregnancy, women felt that the advice required ‘a lot of time to process’ and was also delivered in formats they considered unsuitable:

I’m already stressed with my pregnancy and you’re giving me leaflets and telling me to read here and here… I cannot be asked to read all those things. It’s tedious and I don’t have the time (P6).

Several other women talked about being ‘overloaded’ with dietary information, which they ‘struggled to relate’ to. For example, a participant remembered being advised to eat food that has ‘this or that nutrient’ because ‘they are good for your pregnancy’, but expressed a challenge with identifying which of her common meals had these recommended nutrients:

We eat a lot of our local food here, but we don’t always know which nutrients they have or what they don’t have. When the midwife says eat more… vitamin complex or whatever… I don’t know if my eba and egusi soup has that or not [laughs] (P10).

The participant expresses, here, a lack of knowledge on the nutritional content of the food she normally eats, whereas she needs this information in order to be able to determine the suitability of the food she eats during pregnancy. In another example, a participant received advice on things to avoid during pregnancy, but felt that the examples of food being cited were incongruent with her habitual dietary behaviours:

She was saying I think, like salmon, shark, and some cheese… blue cheese or something. I don’t eat any of them anyways (P2).

In both cases, the advice being given to the women did not take their food context into account, which also means it may not be addressing their actual dietary needs. Most women were unable to remember any specific examples of diet-related advice they had received from midwives, and they could also not remember what advice they had been given relating to nutrient supplements. A majority of the women either reported inconsistent or no use of nutrient supplements during pregnancy, with several stating that they were ‘not really sure’ why they were being asked to take these supplements.

With the lack of diet-related pregnancy advice that suited their needs, participants tended to rely more on the advice they receive from family members and friends, who often ‘take time to explain what is good or not good’ for them during pregnancy, taking into account the types of food they normally eat:

My husband made a list of all the things I always cook—the things we normally eat. Then, he did some research and showed me the ones that I should not eat and the ones that I can eat… or the things I can eat but only in small quantities (P11).

The woman in this example was being advised in the context of her dietary behaviours, which made the advice more practical for her. With this approach, she was able to identify things she normally ate but needed to avoid or limit in pregnancy. Several women said they found the advice given by their mothers, sisters, and friends who had previously been pregnant to be ‘very helpful’, because these were backed by examples from their past experiences, and so ‘you can understand why they tell you to eat some things or not to eat some things’ (P12). Thus, family members and friends were seen as instrumental in shaping women’s dietary behaviours during pregnancy.

(ii) Pregnancy pampering

The disadvantage of women relying on their relatives for dietary advice is that their relatives encouraged the idea of eating for two, because it was believed that ‘a pregnant woman eats for herself and the baby’ (P5). Most women recalled several instances where they had been advised, encouraged, or sometimes ‘forced’ by their relatives to eat more than they felt was necessary, because ‘babies are always hungry’ (P2). Family members, friends, and other external relatives also had the habit of ‘pampering’ pregnant women, e.g., by ‘making sure you always have something to eat’ (P3), ensuring that ‘you always eat what you like’ (P5), ‘cooking for you at 2 a.m.’ (P8), or going out to get their food cravings:

I was about 7 months. I could smell something so nice coming from my neighbour’s house [laughs]… you know pregnancy… this day I got that smell, I wanted the food so bad. I couldn’t resist, so my husband went there and begged the woman for some of her food (laughs) (P8).

This case, though unusual in that it involves the neighbours, is typical in that it shows the extent to which relatives would go to help satisfy pregnant women’s desires or cravings. Participants also described how pregnant women back home were often visited by external relatives, neighbours, and other well-wishers who cooked varieties of food and brought for them on a daily basis. Meanwhile, ‘here in England, you’re on your own’, since most of their relatives were either not in England or ‘here but busy’. However, some women still experienced this pregnancy pampering from their friends and relatives in England, who would offer to take them out to eat, buy them take-out food, or ask them what they were craving for so they could buy/cook and bring.

Pregnancy pampering was also discussed in the context of PA, where relatives—especially mothers, grandmothers, or mothers-in-law—insisted on doing household chores for pregnant women, such as cleaning, cooking their meals, or doing their shopping, ‘because they don’t want you to stress’ (P5). Even in England where participants were mostly separated from their families and no longer had the same levels of community support as they did back home, some women described how their friends or external relatives took time off work to come over to their houses a few months before the baby’s arrival, ‘to cook, clean, and look after you until after the baby is born’ (P3).

While admitting that they enjoyed these forms of treatment because ‘it makes you feel loved’ and ‘it makes the pregnancy less stressful’, the women also felt that it made it difficult for them to resist their pregnancy cravings, especially as they were usually for things that they believed were unhealthy. They also felt it made them less physically active, which they believed was ‘not good’ for them during pregnancy.

(iii) Conflicting information on PA in pregnancy

Midwives were recognised as helpful and giving ‘important advice’ on PA in pregnancy, which women found to be very useful. Participants reported that being in England exposed them to ‘a lot of information’ on the importance of PA during pregnancy and on different forms of exercise they could do during pregnancy. Meanwhile, pregnant women back home were positioned as lacking this information. For example, women previously did not think of walking as a form of PA during pregnancy or that being physically active could be as simple as taking the stairs instead of the lift, going swimming, or going to work while being pregnant.

Participants reported that PA is usually not given much importance during discussions with nurses or midwives back home; hence, ‘many women don’t know that it’s important to do things to make yourself active when you’re pregnant’ (P4). Rather, pregnant women are often provided with ‘discouraging’ advice about PA during pregnancy:

… because we know that pregnancy alone is already stressful… so, pregnant women are not supposed to stress. They say that if a pregnant woman is doing a lot of things, like bending down to wash things, cleaning the house, sweeping, walking for a long distance, it can cause miscarriage or make the baby come before time (P14).

On the basis of their interpretation of stress (which is taken to mean any form of physical exertion or ‘anything that can make you tired’ (P5)), pregnant women are discouraged from engaging in any such activities, with the belief that ‘stress can harm the mother and the baby’ (P1). However, while in England, participants adopted simple PA behaviours during pregnancy, despite still receiving conflicting and often discouraging pieces of advice from their relatives.

Although pregnant women back home received less PA-related advice from health providers, some participants felt that the format in which midwives back home delivered PA advice to pregnant women (e.g., through group dance sessions with pregnant women) was a better approach to encourage women to be active during pregnancy, unlike the one-to-one sessions they had with midwives in England.

### 3.2. Conceptual Framework of Pre- and Post-Migration Influences on Women’s Weight-Related Behaviours

**Figure 1:**
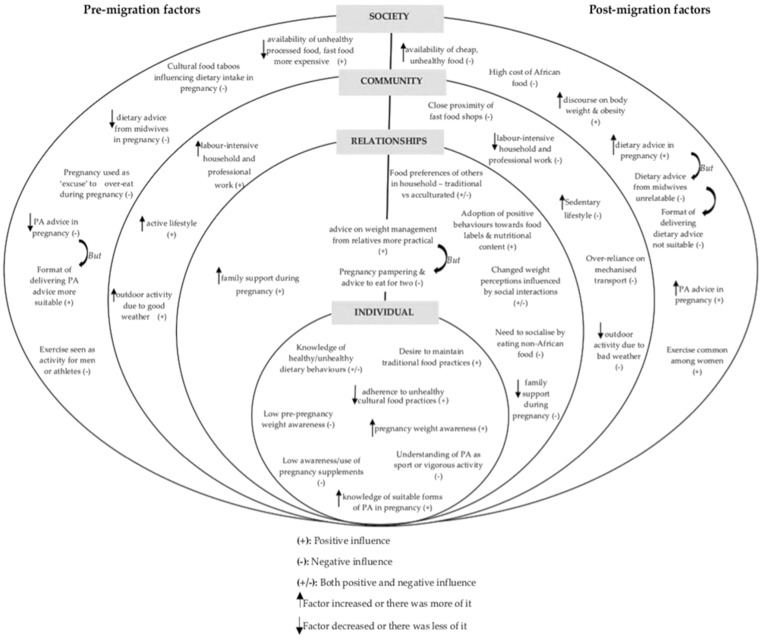
Conceptual framework of pre- and post-migration influences on participants weight-related behaviours (adapted socioecological model).

The pre- and post-migration factors reported in the themes cut across different levels, including individual and interpersonal factors, as well as wider societal and environmental influences. Figure 1 illustrates the interactive influences of these factors, showing how they either served as barriers and/or enablers for having and maintaining healthy weights both before and during pregnancy. The individual level identifies personal factors such as the women’s personal knowledge and perceptions of healthy dietary, PA, and weight management behaviours. The relationships level identifies the influences of close relationships such as family members, friends, and partners on women’s behaviours. The community level shows the influence of settings in which women have social interactions (e.g., their neighbourhoods, places of work or education) and identifies characteristics of these settings that are associated with women’s weight-related behaviours (e.g., proximity of fast food shops, types of work, transportation systems). The society level looks at the broad societal and environmental factors that influence women’s weight-related behaviours, including sociocultural norms and the healthcare system.

## 4. Discussion

This study explored African migrant women’s perceptions of the pre- and post-migration influences on their weight-related behaviours before and during pregnancy, as well as their experiences of weight management support during pregnancy. A key distinctive feature of the conceptual framework used in this study is that it takes into account the complex interplay between pre-migration and post-migration influences on women’s weight-related behaviours and ability to maintain a healthy weight. In addition, cultural factors play an important role in women’s reproductive health, and some of these factors may not be well understood without understanding the background context as it is in their countries of origin.

Comparing influences on women’s weight-related behaviours before and after migration shows some pre-migration factors which served as enablers for having a healthy weight and related behaviours, which were no longer present after migration. For example, back home, women reported being generally more active and that the food environment was less unhealthy, whereas they did not consider this to be the case in England. Several studies have reported on the obesogenic environment in HICs and shown how this contributes to an increased risk of overweight and obesity among migrants, usually through acculturation [31,32,33,34]. However, while migration studies predominantly report negative influences of acculturation on migrant women’s weights and weight-related behaviours, this study also found some positive influences. For example, women reported an increase in their consumption of fruits, vegetables, and water after migration, as well as the development of positive behaviours such as paying more attention to the caloric content of food before purchasing or eating. Similar observations were made in a study conducted in Belgium [35] which showed that, alongside the adoption of unhealthy behaviours, being exposed to the HIC culture resulted in an increase in nutrition knowledge and the adoption of healthier eating behaviours among migrants. Migrating to England also increased women’s exposure to health messages on overweight and obesity, raising their awareness of the risks of excess weight gain and their knowledge about the positive impacts of PA in pregnancy and types of PA suitable for pregnant women. The women also questioned whether some of their previous cultural beliefs and practices were ‘unhealthy’, such as food taboos which may be limiting intakes of important nutrients during pregnancy. To our knowledge, no study has explored the positive influences of acculturation on African migrant women, and it is unclear how different degrees of engagement in and adoption of HIC cultures are associated with their behaviours or weight perceptions. Further studies on acculturation patterns and their associated influences would be useful to identify factors that can facilitate the adoption of healthy weight-related behaviours among African migrants and how to further encourage these.

In addition to health providers who were cited as playing an important role in providing guidance on diet and PA in pregnancy, participants’ relatives were shown to play a major role on their food and weight-related beliefs, as well as in providing social support. Relatives were providing practical dietary advice since the advice from health providers in England did not always meet the women’s needs. Women also had more support during pregnancy from their families while living back home, as caring for pregnant women was a shared responsibility. However, this was no longer the case after women migrated to England. African migrant women have been shown to face challenges during pregnancy due to having reduced social networks after migration [36,37], and studies have reported migrant women feeling isolated, lonely, and depressed [38,39], all of which can negatively influence their weight-related behaviours and pregnancy outcomes [40,41]. Forming collaborations between health providers and relatives of pregnant African migrant women may be helpful towards supporting healthy pregnancies and facilitating the provision of weight management support for pregnant women. Further research with relatives involved in caring for pregnant women could also help identify ways to support them throughout their pregnancy. This can also be helpful in addressing common beliefs around food quantity and PA in pregnancy.

Migrant interactions were shown to have both positive and negative influences on women’s weight-related behaviours. Women felt encouraged or enabled by African peers or relatives in England to maintain their traditional dietary practices; meanwhile, being around non-migrants encouraged the adoption of non-African dietary patterns. However, interactions with other Africans could encourage the idea that African women need to be ‘thick and curvy’, whereas interacting with non-Africans influenced women to want to be fit. These findings highlight some important considerations relating to migrant interactions in host countries and how they may present opportunities for supporting weight management for migrant women, for example, what ideals or behaviours these women are more likely to adopt and what aspects of their social lives can influence these changes.

This study makes several contributions to the literature. NICE guidelines on behaviour change [42] highlight the need to recognise how women’s social contexts and relationships may affect their behaviours, and they also state that effective weight management programmes should identify and address barriers to change, as well as address reasons why some individuals might find it difficult to manage their weights [43]. This study addresses factors that influence weight-related behaviours both before and during pregnancy in African migrant women, and it provides relevant information that can be useful for designing appropriate weight management support interventions for them.

A recommendation for practice from this study relates to the provision of healthcare services for African migrant women, especially when providing support with nutrition and weight management in pregnancy. First, it is important for health providers not to make any assumptions, for example, assuming that everyone understands the healthcare system or that a single approach to providing dietary advice would be suitable for all women. The healthcare system in England is different from what the women had experienced in their countries of origin, and such differences can influence their perceptions of what they need, their healthcare-seeking behaviours, and their use of these services. It is also important to acknowledge how cultural factors can influence their diet and PA behaviours and engage in meaningful dialogue with them about these, to enable them to express their unique challenges. There is a common stereotype—both in the general society and in the medical field—that Black women are strong and can handle anything [44,45]. In other words, Black women are required to respond to difficult situations by portraying strength and concealing distress [45]. While typically intended as a compliment, such stereotypes may prevent women from expressing when they need support. Evidence of vulnerability was apparent when conducting interviews with participants, especially when they talked about their challenges with making healthy food choices during pregnancy. These women can be better supported by appreciating their unique challenges, listening to their concerns, and providing individualised support. Further studies exploring women’s pregnancy experiences when they have just arrived in the UK compared to subsequent pregnancies after having lived in the UK for some years could also be helpful in identifying factors that they found useful in enabling them to manage their pregnancy and weight-related needs.

A key strength of this study is that interpretations of resulting codes and themes were reviewed and discussed throughout by research team members, to ensure that findings were an accurate representation of participants’ views and supported by the literature. The principal investigator shared ethnic and cultural similarities with participants, which was helpful in establishing rapport and enabling the women to be more open about their experiences [46]. While acknowledging that this could have influenced the interpretation of data due to preconceived ideas or experiences, this was mitigated by the rigorous data analysis approach used, which involved researchers from different backgrounds. There are some limitations that may influence the interpretation of findings from this study. First, as is usually the case with qualitative studies, the interviews were reliant on participants’ ability to recall and recount details about their lives, circumstances, and behaviours. Secondly, some important data may have been lost when defining themes to focus on salient and recurrent issues. However, despite these limitations, this study provides useful insights into African migrant women’s perceptions of the pre- and post-migration influences on their weight-related behaviours before and during pregnancy.

## 5. Conclusions

This study identified pre- and post-migration influences on African migrant women’s weight related behaviours, and it outlines how factors such as culture, societal norms, and social interactions, both in their countries of origin and in England, influence weight preferences and weight management behaviours before and during pregnancy. Both positive and negative influences of migration on women’s weight and related behaviours were identified, highlighting areas for improvements in providing weight management support; further exploration is needed of how these influences may contribute towards the women’s multicultural identities in the context of weight management.

## Figures and Tables

**Table 1:** Participant characteristics.

Characteristic	Total Number of Participants (*n* = 23)
Age, mean (range)	30.7 (23–41)
Country of origin, *n*	
Nigeria	9
Cameroon	8
Ghana	6
Duration of residence in England, *n*	7 months–22 years (mean 6.8 years)
Total number of children, *n*	
1	5
2	9
3 or more	6
Pregnant at time of study, *n*	5
Time since last birth, *n* Less than 6 months 6–11 months 1–2 years Over 2 years	6 3 5 4
Level of education	
Up to secondary or pre-university education	7
Attained education at university level	16
Married or cohabiting, *n*	19
Employed, *n*	17
Region of residence, *n* North and Northeast (five different towns and cities) Greater London South and Southeast (4 different towns and cities) West Midlands (five different towns and cities)	6 5 5 7

## Appendix A. Topic Guide Used for Interviews

Interview Questions 1. Changes in dietary behaviours after migration How would you describe the types of food available in the UK and what you eat, compared to when you were back home? Potential areas to discuss further: - Current dietary behaviours - Compare England vs. back home - Changes in dietary patterns after migration - Factors associated with these changes 2. Changes in PA behaviours after migration How would you describe your PA levels in the UK compared to when you were back home? Potential areas to discuss further: - Current PA behaviours - Compare England vs. back home - Changes in PA behaviours after migration - Factors associated with these changes 3. Dietary behaviours during pregnancy Did you make any changes to your usual diet during pregnancy? Potential areas to discuss further: - Any changes to their diet when pregnant compared with before pregnancy - Types of food they ate/avoided - Factors influencing dietary changes during pregnancy - Reasons why they did or did not change their dietary behaviours in pregnancy - Any difficulties with maintaining healthy diet - Compare England vs. back home - If pregnant in African country previously—explore any differences between dietary behaviours in the UK and back home - If not pregnant in African country previously—explore their perceptions on whether their behaviours would have been different if they were back home 4. PA behaviours during pregnancy Did you make any changes to your usual daily activities during pregnancy? Potential areas to discuss further: - Any changes to their PA behaviours when pregnant compared with before pregnancy - Types of activities they did - Factors influencing dietary changes during pregnancy - Reasons why they did or did not change their PA behaviours in pregnancy - Any difficulties with staying active during pregnancy - Compare England vs. back home - If pregnant in African country previously—explore any differences between PA behaviours in the UK and back home - If not pregnant in African country previously—explore their perceptions on whether their behaviours would have been different if they were back home 5. Perceptions on pre-pregnancy weight and weight status in pregnancy There are several things that could have an effect on a woman or her child during pregnancy, things like smoking, drinking alcohol, taking drugs, being overweight or obese before pregnancy, etc. Are there any of these things that you think are very important to look out for? Potential areas to discuss further: - Which pregnancy risk factors they give priority to and why - Perceptions about their weights before vs. during pregnancy - How they think their pre-pregnancy weight could affect their pregnancy - Whether they think of overweight/obesity as a risk factor in pregnancy - Compare England vs. back home 6. Weight management advice in pregnancy When you were pregnant, did you ever have any discussions about the risks of excess weight gain in pregnancy? Potential areas to discuss further: - Diet or PA-related information received during pregnancy - Source(s) of information - Their views on the information they received - How relevant the information was for them - Advice and sources found to be most helpful/useful and reasons - Compare England vs. back home - If pregnant in African country previously—explore any differences between information received in the UK and back home - If not pregnant in African country previously—explore their perceptions on whether the information would have been different from that received in the UK
